# Systems-based proteomics to resolve the biology of Alzheimer’s disease beyond amyloid and tau

**DOI:** 10.1038/s41386-020-00840-3

**Published:** 2020-09-08

**Authors:** Sruti Rayaprolu, Lenora Higginbotham, Pritha Bagchi, Caroline M. Watson, Tian Zhang, Allan I. Levey, Srikant Rangaraju, Nicholas T. Seyfried

**Affiliations:** 1 Department of Neurology, Emory University School of Medicine, Atlanta, GA 30322 USA; 2 Center for Neurodegenerative Disease, Emory University School of Medicine, Atlanta, GA 30322 USA; 3 Department of Biochemistry, Emory University School of Medicine, Atlanta, GA 30322 USA

**Keywords:** Biochemistry, Neuroscience

## Abstract

The repeated failures of amyloid-targeting therapies have challenged our narrow understanding of Alzheimer’s disease (AD) pathogenesis and inspired wide-ranging investigations into the underlying mechanisms of disease. Increasing evidence indicates that AD develops from an intricate web of biochemical and cellular processes that extend far beyond amyloid and tau accumulation. This growing recognition surrounding the diversity of AD pathophysiology underscores the need for holistic systems-based approaches to explore AD pathogenesis. Here we describe how network-based proteomics has emerged as a powerful tool and how its application to the AD brain has provided an informative framework for the complex protein pathophysiology underlying the disease. Furthermore, we outline how the AD brain network proteome can be leveraged to advance additional scientific and translational efforts, including the discovery of novel protein biomarkers of disease.

## Introduction

Alzheimer’s disease (AD), the most common cause of dementia worldwide, is characterized by progressive declines in cognition and everyday function [1]. An estimated 40 million people across the globe have AD and, due to extending lifespans, this number is only expected to increase [1, 2]. Therefore, without effective disease-modifying therapies, AD poses a uniquely devastating threat to the health and welfare of the elderly.

For the past several decades, AD research and biomarker development have centered around two proteins, amyloid-beta (Aβ) and tau. A wealth of evidence suggests these proteins play a pathogenic role in disease. Aβ, which begins accumulating over a decade prior to clinical symptoms, comprises the cortical extracellular plaques characteristic of AD [3]. In addition, gene mutations that directly enhance Aβ aggregation underlie rare familial forms of the disease [4, 5]. Meanwhile, tau accumulation as intracellular neurofibrillary tangles (NFTs) is another hallmark feature of AD and strongly associated with progressive neuronal loss and cognitive decline [3]. For these reasons, a definitive AD diagnosis requires the postmortem presence of both Aβ and tau pathologies in the cerebral cortex [6]. Antemortem diagnosis also relies heavily on the presence of Aβ and tau, either as measured in the spinal fluid or imaged using positron emission tomography (PET) [7–10].

However, mounting evidence indicates that Aβ and tau represent only a fraction of the complex and heterogeneous biology of AD. Large clinical trials of Aβ-targeting therapies have repeatedly failed, suggesting that amyloid-centric therapy is insufficient to quell disease [11]. Simultaneously, a growing number of genetic, cellular, and biochemical studies have linked AD pathogenesis to a more diverse array of biological mechanisms involving a variety of cell types. Thus, some have postulated that AD maintains two pathogenic phases, including a proteopathic “biochemical” phase characterized by the direct toxic effects of Aβ and tau aggregation and a “cellular” phase encompassing a web of complex feedback and feedforward cell-mediated mechanisms that promote irreversible degeneration [12]. This pathogenic framework argues that the dynamic interaction of both phases is critical for disease progression and cognitive decline.

The growing complexity of AD pathogenesis has highlighted the need for additional biomarkers that fully reflect underlying pathophysiology and effectively promote advancements in diagnostic profiling, disease monitoring, and treatment. In response to this urgent need for biomarker discovery, the Accelerating Medicines Partnership (AMP)-AD initiative was launched in 2014. This multidisciplinary effort between the National Institutes of Health (NIH), academia, and industry aims to leverage systems-based scientific strategies to better characterize AD pathophysiology and identify effective therapeutic targets [13]. Amidst this partnership, unbiased network-based proteomics has emerged as a powerful tool for unraveling the intricate biology underlying AD. In this review, we discuss recent large-scale, multidimensional, network proteomic analyses of the AD brain and the applications of these results to additional scientific and translational efforts.

## Expanding AD pathogenesis beyond amyloid and tau

The “amyloid hypothesis” proposes that AD is caused by a linear series of events initiated by Aβ and culminating in progressive neuronal loss and cognitive decline [14]. Though never universally accepted, this hypothesis remained the most prominent theoretical framework for understanding AD for over two decades. However, the repeated failures of Aβ-targeting drug trials have recently bolstered critics of this hypothesis and broadened investigations of AD pathophysiology. Increasing evidence now suggests that AD features a diverse non-linear pathogenic landscape encompassing a variety of cellular mechanisms with equally prominent roles in disease progression [12].

### The amyloid hypothesis controversy

Aβ peptide is the main component of extracellular plaque pathology in the AD brain [15]. Aberrant Aβ deposition begins over a decade before the onset of clinical symptoms and spreads diffusely throughout the cerebral cortex [16, 17]. According to the “amyloid hypothesis”, progressive Aβ accumulation triggers a cascade of detrimental processes, including intracellular NFT formation, synaptic dysfunction, neuronal death, and finally irreversible dementia [18, 19]. This hypothesis gained favor in the 1990s after several gene mutations directly responsible for aberrant Aβ accumulation (*APP, PSEN1, PSEN2*) were found to underlie rare familial forms of early-onset AD [20–22]. These genetic findings led to several transgenic mouse models that successfully recapitulated Aβ plaque formation, increasing support for the hypothesis (Table 1) [23–29]. Though familial AD cases account for <5% of the AD population, this amyloid-centric framework was promptly extended to sporadic late-onset AD (LOAD) given its identical hallmark pathology [3]. Subsequent genetic association studies of LOAD bolstered this framework, identifying risk factors such as *APOE* that modulate Aβ processing, trafficking, and clearance [19, 20, 30]. This was followed by evidence indicating that soluble oligomeric Aβ mediates synaptic dysfunction and neuronal toxicity [31–33]. For these reasons, enormous research efforts were expended toward Aβ-targeting drug therapies that either inhibited production or facilitated the removal of Aβ from the brain [11].

**Table 1:** Representative transgenic mouse models commonly used to study Alzheimer’s disease (AD).

Model name	Gene(s)	Mutation(s)	Promoter	Background	Availability	Ref.
J20	huAPP770	APP-Swe, Ind	*PDGFB*	C57BL/6	The Jackson Laboratory: Stock No. 34836-JAX	[29]
TgCRND8	huAPP695	APP-Swe, Ind	Ham *Prnp*	Hybrid C3H/He-C57BL/6	Peter St. George-Hyslop	[193]
Tg-SwDI	huAPP770	APP-Swe, Dut, Iowa	Mo *Thy1*	C57BL/6	The Jackson Laboratory: Stock No. 34843-JAX	[194]
APP23	huAPP751	APP-Swe	Mo *Thy1*	C57BL/6	The Jackson Laboratory: Stock No. 030504	[26]
Tg2576	huAPP695	APP-Swe	Ham *Prnp*	C57BL/6 x SJL; C57BL6	Taconic: Stock No. 1349	[195]
APPPS1	huAPP, huPSEN1	APP-Swe; PS1-L166P	Mo *Thy1*	C57BL/6J	Mathias Jucker	[196]
APPswe/PSEN1dE9 (line 85)	mo/huAPP695, huPSEN1	APP-Swe; PS1∆E9	Mo *Prnp*	C57BL/6; C3H	The Jackson Laboratory: Stock No. 034829	[197]
5xFAD	huAPP, huPSEN1	APP-Swe, Flo, Lon; PS1-M146L, L286V	Mo *Thy1*	C57BL/6 x SJL; C57BL6	The Jackson Laboratory: Stock No. 034840; Stock No. 034848	[198]
JNPL3	huMAPT 4R0N	P301L	Mo *Prnp*	C57BL/6, DBA/2, SW mixed	Taconic: Stock no. 2508	[199]
hTau.P301S	huMAPT 4R0N	P301S	Mo *Thy1.2*	CBAxC57BL/6	Michel Goedert; LifeArc	[200]
PS19	huMAPT 4R1N	P301S	Mo *Prnp*	C57BL/6 x C3H	The Jackson Laboratory: Stock No. 008169	[201]
3xTg-AD	huAPP, moPsen1, huMAPT 4R0N	APP-Swe; MAPT-P301L; *Psen1* M146V knock-in	Mo *Thy1.2*	C57BL/6;129	The Jackson Laboratory: Stock No. 34830-JAX	[202]

Although this is not a thorough list of mouse models, Jankowsky and Zheng [203] and www.alzforum.com/research-models provide detailed lists of transgenic and non-transgenic mouse models. Mutations: Swe = Swedish (M670/671NL); Flo = Florida (APP I716V); Lon = London (APP V717I), Ind = Indiana (V717F); Dut = Dutch (E693Q); Iowa (D694N).

*APP* amyloid precursor protein, *PSEN1/PS1* presenilin 1, *hu* human, *mo* mouse, *Ham* hamster.

Yet, the repeated failures of these drugs have brought the amyloid hypothesis under fire [19, 31, 34]. While many argue that these trial failures are due to poor study design or patient selection, critics of an amyloid-centric framework maintain that Aβ is simply not the key to disease pathogenesis. Its tendency to accumulate for years without causing symptomatic disease (i.e., asymptomatic AD or AsymAD) supports this conclusion [35, 36]. In one notable case, even harboring an autosomal dominant *PSEN1* mutation was insufficient to cause overt dementia [36]. Moreover, the growing recognition of overlapping pathologies (e.g., Lewy body inclusions, TDP-43 pathology, vascular lesions, etc.) among dementia patients indicates a wide range of biological heterogeneity within AD that necessitates a broader approach to scientific investigations of its pathophysiology [37–39].

### The evolving role of tau

Hyperphosphorylated tau accumulates as intraneuronal NFTs, a hallmark feature of AD pathology [3]. Though the amyloid hypothesis places NFTs downstream of Aβ deposition, increasing evidence suggests that aberrant tau accumulation is a complex multifactorial process that can occur independent of amyloid. Such observations date back to the pathological descriptions of Heiko and Eva Braak in 1991, who found that AD-related tau deposition followed a stereotypical pattern of progression regardless of variations in Aβ distribution [40]. Subsequent studies identified the locus coeruleus as the first site of pathology in AD subjects, with NFTs preceding cortical tau or Aβ deposits [41–43]. More recently, Crary et al. coined the term “primary age-related tauopathy” (PART) after observing that the postmortem brains of many elderly individuals harbor NFTs indistinguishable from those of AD but in the absence of Aβ plaques or cognitive decline [44]. Furthermore, aggregated hyperphosphorylated tau is a well-described pathological hallmark of several other non-AD disorders, such as frontotemporal lobar degeneration (FTLD) [45]. Together, these findings argue against tau accumulation as a linear consequence of Aβ deposition.

As many critically question the amyloid hypothesis, tau has begun to receive more attention as a potential therapeutic target. Compared to amyloid burden, tau levels correlate much more strongly to cognitive symptoms, suggesting a more direct link to disease progression [46]. However, the efficacy of these tau-targeting strategies has yet to be demonstrated. In many instances, success in animal models has not translated into cognitive benefits in humans. Clinical trials of methylene blue-derived tau inhibitors in AD have generally yielded disappointing results [47–49]. Select tau immunotherapies currently employed in AD trials have previously failed to demonstrate any effect on disease progression in the primary tauopathy progressive supranuclear palsy (PSP), fueling reservations about their utility in other neurodegenerative disorders [49]. Indeed, many remain skeptical that targeting tau alone will be the key to successful AD modification, especially given the increasing evidence of other cell-mediated pathogenic processes. Nevertheless, numerous tau-targeting trials are currently on-going, tackling a variety of mechanisms that contribute to tauopathy development. The next several years should reveal much more about the utility of this approach and in turn, the role of tau in AD pathogenesis.

### The cellular phase of AD

In a 2016 review, De Strooper and Karran discuss the overwhelming evidence for a “cellular phase” of AD, a decades-long period of complex feedback and feedforward mechanisms between neurons, glia, and the endothelium that ultimately precipitates the irreversible damage underlying cognitive decline [12]. In this framework, AD begins with a “biochemical phase”, characterized by aberrant amyloid precursor protein (APP) processing, Aβ accumulation, and tau hyperphosphorylation. These biochemical changes exert proteopathic or “aggregate” stress on surrounding tissues, sparking a cell-mediated phase of disease. Initially, this “cellular phase” maintains homeostasis amidst the growing proteopathic disruption. However, as the diseased individual ages, these compensatory cellular mechanisms evolve into increasingly dysfunctional and detrimental processes. Defective Aβ clearance, mediated by the brain’s perivascular circulation and glymphatic system, is considered one of the first events in this biochemical to cellular transition [12]. Dysfunction of the astrocytes and specialized endothelium comprising this clearance system not only contributes to increasing amyloid accumulation but also promotes irrevocable disruption of other neurovascular and glioneuronal functions, including lipid metabolism, myelin turnover, and immune regulation. Cell-mediated feedback and feedforward mechanisms allow this dysfunction to thrive independently of accumulating amyloid and tau, precipitating chronic inflammation and circuitry imbalances that result in cell failure and death.

It is this cellular transition, argue De Strooper and Karran, that propels the onset of cognitive symptoms. Thus, the hallmark proteinopathy of AD does not directly cause symptomatology but instead serves as an upstream trigger for destructive cellular processes. There is strong evidence to support the causative role of cell-mediated dysfunction in the pathogenesis of AD. Several genes encoding proteins intimately associated with the endothelium and Aβ clearance are highly validated AD risk factors, such as * APOEε4, PICALM*, and *CLU* [50, 51]. Likewise, genome-wide association studies (GWAS) have identified several single-nucleotide polymorphisms (SNPs) in immune genes as independent risk factors for LOAD [52–55], directly implicating microglia-mediated mechanisms in sporadic AD progression. In addition, two groups of investigators independently identified variants of the microglial *TREM2* gene that increase susceptibility to LOAD with an odds ratio similar to that of *APOEε4* [56–58]. These genetic findings are further supported by the common observation of aberrantly activated microglia in pathologically relevant AD brain regions, including within and surrounding Aβ plaques [59, 60].

The wealth of evidence indicating the causative role of these cellular processes in AD pathogenesis strongly supports a more holistic approach to AD investigation and biomarker discovery. In the remainder of the review, we discuss how global network-based proteomic approaches to the AD brain have helped characterize these cellular changes, their roles in disease, and associated protein biomarkers. This unbiased strategy does not ignore or discount the proteopathic biochemical phase, but instead puts this protein accumulation and its direct effects into the larger context of neurovascular and glioneuronal dysfunction.

## A global network proteomic approach to the AD brain

The expanding framework of AD pathophysiology has necessitated more holistic network-based “-omic” approaches to scientific investigation. The multidisciplinary AMP-AD initiative has sprung to the forefront of these systems-based efforts, leveraging over 2000 brain tissues across its participating institutions to perform large-scale integration of network genomic, epigenomic, RNAseq, and proteomic data [13]. Under this initiative, network proteomic analysis has emerged as a valuable tool for assessing pathophysiological changes in both AsymAD and later stages of disease. This approach organizes complex proteomic data into unbiased groups or “modules” of protein co-expression that reflect various molecular, cellular, and circuit-level phenotypes.

This shift toward systems-level proteomic analysis mirrors efforts in AD transcriptomics, a field that has employed similar network strategies to contextualize the many genetic variants now associated with LOAD [61]. Yet, protein-level analyses have demonstrated disease-related alterations not readily measured in transcriptomic networks. Only 30–40% of modules in the AD brain network proteome overlap with those of the network transcriptome [62, 63]. While differential protein expression within these overlapping modules is reasonably concordant (R~0.5), we have repeatedly observed targets among these modules with highly discordant changes at the protein and RNA levels [62–64]. Furthermore, only half of the disease-related variance observed in the AD network proteome is reflected in gene expression at the transcriptome level, the remainder representing post-transcriptional and post-translational effects [64]. These findings, consistent with previous comparisons of protein and mRNA data [65], strongly support the utility of protein profiling in AD and its complementarity with transcriptomic studies. In the remainder of this section, we will discuss the mass spectrometry-based pipelines and computational methods used to conduct global network proteomic analyses of human AD brain tissue.

### Strategies for mass spectrometry-based quantification

**Fig. 1:**
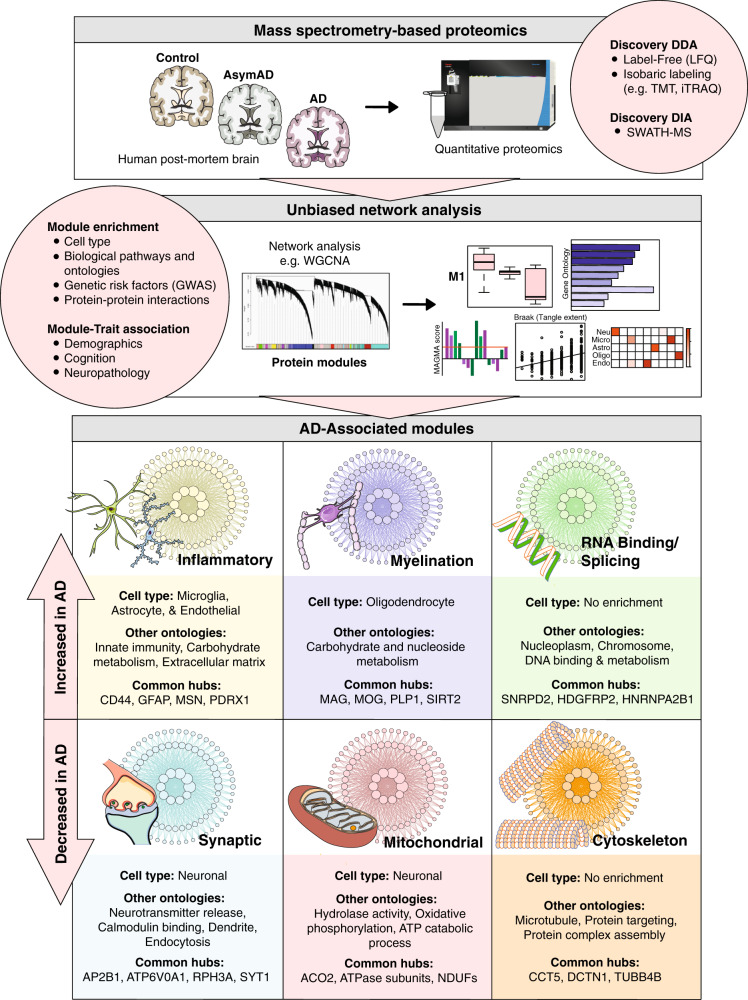
Unbiased network-based proteomics to characterize the complex biochemical and cellular endophenotypes of Alzheimer’s disease (AD). Traditional “bottom-up” mass spectrometry (MS)-based techniques, such as label-free quantitation (LFQ) or isobaric labeling, are used for protein identification and quantification in a large cohort of postmortem brain tissues from control, asymptomatic AD (AsymAD), and AD cases. Following fractionation, the deep discovery proteomic data generated from these large cohorts are organized into biologically meaningful groups, or modules, of proteins with sophisticated analytical methods, such as weighted gene correlation network analysis (WGCNA). The co-expression modules are assessed for enrichment with specific cell types, organelles, biological pathways, and genetic risk factors generated from genome-wide association studies (GWAS). In addition, abundance changes at the module level can be correlated with disease status, clinical features, and neuropathological measures. Using this framework, six core and highly conserved modules across AsymAD and AD cohorts with reproducible links to specific cell types, organelles, and biological functions have been identified. Three of the modules are consistently increased in the AD brain network proteome: inflammatory, myelination, and RNA binding/splicing, while the remaining three are consistently decreased: synaptic, mitochondrial, and cytoskeleton.

Network proteomic analyses of the AD brain have relied heavily on traditional “bottom-up” mass spectrometry (MS)-based techniques for protein identification and quantification (Fig. 1). This workflow typically comprises enzymatic protein digestion (e.g., trypsin) followed by liquid chromatography (LC) separation and tandem MS (MS/MS) measurement of peptides. Data-dependent acquisition (DDA) is commonly utilized, in which a limited number of precursor peptides are stochastically selected in the first stage of MS (MS1) to be fragmented and analyzed in the second tandem stage (MS2). These quantified peptides are subsequently identified by spectral matching and analyzed using well-validated statistical and bioinformatic strategies [66–71]. This workflow has proven robust and accurate in its protein profiling of complex mixtures, explaining its popularity among MS-based approaches.

A variety of technological strategies have further enhanced this workflow, bolstering the quantification and depth of resulting proteomic datasets. For example, there has been an increasing transition from label-free to isobaric-labeling approaches. In label-free quantitation (LFQ), each sample is prepared and analyzed individually by LC-MS/MS. Though MS1 peptide selection is biased toward the most intense signals [72], the inherently stochastic nature of DDA impacts the consistency of peptides chosen and analyzed across samples. Variability in mass measurements and LC peptide retention times may also introduce inconsistencies in precursor peptide selection and quantification. This creates a well-described “missing value” problem that ultimately limits the number of quantifiable proteins in a given LFQ dataset [72–75]. Multiplex isobaric peptide labeling, as with tandem mass tags (TMTs) and isobaric tags for relative and absolute quantitation (iTRAQ), helps to mitigate the issue of missing values by enabling the analysis of multiple samples simultaneously within a single LC-MS/MS analysis, as many as 16 with TMT [76, 77]. This strategy, when coupled to off-line fractionation, can result in the quantification of thousands of additional proteins and has permitted remarkably deep proteomic analysis of AD brain tissue [74, 75, 78, 79]. Johnson et al. demonstrated this advantage in one of the first TMT-MS network proteomes of the AD brain [74], which quantified 6533 proteins across 47 brain tissues compared to just 2736 proteins quantified by LFQ-MS when applied to the same samples. However, despite these achievements in quantification, TMT-MS may still yield missing values across multiplexed batches when analyzing large numbers of samples [80].

To better address the problem of missing values, data-independent acquisition (DIA) has emerged as an alternative to the stochastic nature of DDA proteomics. Instead of selecting a limited number of MS1 peptides for analysis, DIA performs MS2 quantification on the entire MS1 spectrum. Therefore, DIA results in the identification of nearly all detectable peptides within a selected mass range, allowing for comprehensive and accurate quantification of identified proteins in the sample with minimal missing values [81, 82]. Due to complex chimeric mixtures of MS2 spectra, DIA data is more difficult to analyze compared to DDA spectra and typically requires more sophisticated computation. Yet, as DIA technology advances, such roadblocks to its widespread use in discovery-driven analyses will likely recede. For instance, sequential window acquisition of all theoretical mass spectra (SWATH-MS), a relatively user-friendly technology capable of deep proteome coverage and high quantitative accuracy [72, 83], stands to significantly advance the application of DIA to complex tissue analyses. Efforts to adapt this strategy for the quantification of bulk brain proteomes are already underway [84]. Meanwhile, targeted DIA approaches, such as selected or multiple reaction monitoring (SRM/MRM) and parallel reaction monitoring (PRM), have already become widely used in research settings for more robust quantification of pre-specified individual peptides and post-translational modifications (PTMs) [81]. The use of these targeted approaches in the validation and clinical translation of discovery-driven data is discussed later.

### Principles of network building and module identification

Organizing such deep discovery proteomic datasets into modules of protein co-expression requires well-validated statistical algorithms. Widely validated in transcriptomic studies, weighted gene correlation network analysis (WGCNA) has been the statistical algorithm of choice among AD proteomic network analyses [61, 62, 85–87], though various alternatives are rapidly emerging [87, 88]. Using graph theory principles, these algorithms identify modules of proteins with highly correlated abundance levels across samples. The protein co-expression within each module may be driven by a variety of biological, physiological, and/or technical factors. Thus, it is important to comprehensively investigate individual module characteristics (Fig. 1). First, assessing connectivity within each module can identify module-specific hubs, or those proteins that are most central to module function [61]. In addition, co-expression modules can be analyzed for enrichment with markers of specific cell types, organelles, biological pathways, and genetic risk factors. Finally, abundance changes at the module level, often reported as eigenproteins or average expression levels, can be correlated with disease status, clinical features, and neuropathological measures. In this way, differential protein expression within a complex mixture can be effectively contextualized within the global biochemical and molecular pathways driving disease.

Network analyses of AD brain tissue demonstrate the most reproducibility at the module level, followed by the hub protein level, and then finally at the level of precise protein connectivity rankings and module protein membership [61, 89–94]. To obtain module-level reproducibility, 20 independent samples are typically considered adequate; however, systematic consistency at the more granular hub and module membership levels may require hundreds of samples [61, 93, 94]. In a typical proteomic pipeline, protein abundances are regressed for effects of age, sex, and postmortem interval (PMI) prior to building the network, but this decision will often depend on the goals of the analysis. Batch correction for technical variance can be performed using a variety of statistical approaches, each with its own strengths and weaknesses depending on the nature of the expression data [95, 96].

Module-level abundance profiles are often statistically correlated to various phenotypic traits of disease, such as amyloid burden, tangle deposition, and cognitive decline. These module-trait correlations indicate those protein groups with strong positive or inverse relationships to disease. Module-enrichment profiles can also offer important insights into proteomic composition. These analyses are achieved using well-validated reference databases and are designed to detect the over-representation of module proteins with known cell type, biological, or genetic risk factor associations. Cell type enrichment is typically performed by cross-referencing module proteins with marker lists derived from existing reference proteomes or transcriptomes of purified murine brain cells [62, 97, 98], although single-cell human datasets also hold promise in this regard [99]. Meanwhile, various resources exist for pathway and ontology analysis [100–102], such as GO-Elite. This flexible analytical tool allows users to incorporate a variety of reference and custom databases to examine ontological over-representation at the biological, molecular, and organellar levels [101]. Finally, genetic risk factor enrichment is performed using integrative algorithms like Multi-marker Analysis of GenoMic Annotation (MAGMA) [103], a powerful gene-set analysis tool that allows for module integration with AD GWAS data [62, 64, 74, 96]. Modules enriched with AD risk factors are considered reflective of causal, rather than reactive, disease mechanisms.

### Limitations of proteomic analysis in human brain tissue

Despite a variety of technological advancements, proteomic analysis of bulk human brain tissue has its limitations. Perhaps the biggest challenge is the inter- and intra-regional variability found throughout brain tissue. Due to differences in neurodegenerative vulnerability, one cortical region rich in disease pathology may not reflect the protein pathophysiology of a relatively spared distant region. Intra-regional tissue heterogeneity in cell type densities or pathological burden may also yield poorly representative results. These challenges necessitate large-scale consensus proteomic comparisons involving multiple brain regions to draw confident conclusions regarding global findings. As discussed in the next section, collaborative efforts, such as AMP-AD, and advances in high-throughput proteomics have allowed the field to overcome many of these challenges and identify a systems-based proteomic organization conserved across brain samples from a variety of different AD cohorts and cortical regions.

While the network proteomic analysis of bulk tissue can provide an invaluable view of global cell type-specific changes in disease, this approach is limited in examination of the cellular microenvironment and its effects on protein levels. Cell populations display a wide dynamic range of protein concentrations driven by their immediate surroundings. Bulk tissue analysis averages these variations in microenvironment and their contributions to protein levels. In addition, reference data for cell type-specificity, derived from healthy brain tissues, may not fully reflect disease-related shifts in cellular protein expression. As detailed later, cell type-specific proteomics can combat many of these limitations by resolving individual communities of glial, endothelial, and neuronal cells and examining the protein expression of each cell population relative to disease state, brain region, and local microenvironment. Such studies promise to uncover the biological subtleties of the cellular phase not readily apparent in the global AD proteome.

Finally, one major limitation of discovery-driven bulk tissue proteomics is its inability to directly probe disease mechanisms. Indeed, additional molecular studies will be required to deduce the biological mechanisms underlying module changes and the precise roles of these protein communities in AD. Yet, a variety of analytical strategies commonly employed in network proteomics may offer valuable insights that effectively guide these additional studies. For example, genetic risk factor enrichment can identify those modules more likely to have causative, rather than reactive, roles in disease and support further investigations into the potential disease-related mechanisms associated with these protein communities. The identification of modules altered in AsymAD also provides a window into the earliest drivers of AD and may help guide the mechanistic evaluation of preclinical disease. Furthermore, as discussed below, the application of network approaches to protein interactomes has helped characterize key aggregation mechanisms underlying the biochemical phase of AD.

## Organization of the AD brain network proteome

Nearly a dozen informative network-based analyses of the AD proteome have been performed in the human brain [62–64, 74, 78, 96, 104–108], including the dorsolateral prefrontal cortex (DLPFC), temporal cortex, and other cortical regions heavily affected in AD. Using high-throughput MS techniques, these studies have revealed highly reproducible modules across the AD proteome, several of which consistently demonstrate strong correlations to AD diagnosis, Aβ burden, and other phenotypic traits of disease. Alterations in these disease-associated modules are strongly conserved across cohorts and cortical regions, allowing for the recent construction of a large consensus AD brain network derived from hundreds of brain samples [96]. Cell type-specific perturbations appear to drive many of these disease-associated network changes with glia-enriched modules most increased in AD and neuronal modules most decreased in disease (Fig. 1). Yet, these network studies have also yielded AD-associated modules with no apparent links to specific cell types, suggesting these protein changes reflect the biochemical phase of AD. The following sub-sections discuss several of the most highly conserved modules of the AD brain network proteome and the insights they provide into disease. Of note, these consensus observations have been largely regressed for age, sex, and postmortem interval (PMI). Given the potential sex differences in AD, the AMP-AD consortium recently performed a large-scale analysis of sex influences on proteomic data and found no statistically significant contribution of sex to protein module alterations [96], though this remains an active area of study.

### An early anti-inflammatory glial response

Microglia and astrocytes, glial cell populations that mediate the innate immunity and inflammatory responses of the central nervous system (CNS), have been linked in numerous studies to AD pathogenesis. Microglia are phagocytic immune cells that comprise ~10% of adult CNS cells with density varying across brain regions [109–112]. Variants of the microglial *TREM2* gene have been found to increase the risk of sporadic AD by  approximately 3- to 5-fold [56, 57]. Moreover, emerging evidence suggests that *APOE*, a potent immune modulator and strongest of genetic risk factors for LOAD [113–115], may regulate neurodegeneration in a *TREM2*-mediated fashion [116, 117]. Meanwhile, astrocytes are known components of many processes implicated in AD, including synaptogenesis, lipoprotein metabolism, and blood–brain barrier (BBB) regulation [12]. In the AD brain, astrocyte populations undergo a series of pathophysiological changes characterized by hypertrophy, proliferation, and increased expression of intermediate filaments. This “reactive” astrogliosis can be found throughout AD progression, including in early prodromal phases of disease.

Whether this heightened inflammation serves a primarily protective or detrimental purpose in AD remains a central question of disease pathogenesis. *TREM2, APOE*, and other microglial-expressed genes are thought to have protective, homeostatic roles in disease [118]. Yet, single-cell RNA sequencing, which enables the profiling of individual microglial cells with high-throughput datasets, has revealed significant functional heterogeneity among microglial subtypes and their inflammatory profiles in the neurodegenerative setting [109, 119]. Similarly, data examining the effects of microglia and inflammatory mediators on Aβ deposition in APP transgenic mice are mixed, with some studies demonstrating reduced and others exacerbated Aβ accumulation [120–122]. Indeed, it is becoming increasingly clear that glial-mediated inflammation is likely a complex process featuring a variety of disease-associated anti-inflammatory and pro-inflammatory components.

Network proteomics has provided insight into the complexity surrounding AD inflammation, supporting a predominantly protective, anti-inflammatory glial response in the earliest stages of disease. Multiple network proteomes have resolved a large module enriched with microglial and astrocytic proteins that demonstrates progressively increasing abundance levels throughout AsymAD and AD. This highly conserved module features hub proteins implicated in neuroprotection, such as the membrane receptor-cytoskeleton cross-linking protein moesin (MSN) [62–64, 96]. Its activation in association with proteins ezrin and radixin has been implicated in the neuroprotective non-amyloidogenic processing of APP [123, 124]. Peroxiredoxin 1 (PRDX1), another hub of this module, is a family member of antioxidant enzymes also implicated in protective cellular responses [125]. These module associations with neuroprotection are not only limited to hub proteins. In a recent network analysis comprising nearly 500 control, AsymAD, and AD brain tissues (DLPFC, precuneus, temporal cortex), Johnson et al. demonstrated enrichment of anti-inflammatory, neuroprotective markers throughout the MSN module [96]. In a comparison with prior proteomic studies, the authors also found several of these module proteins are decreased in rapidly progressive AD compared to sporadic disease, suggesting that a failure to activate this module may lead to more aggressive cognitive decline [96, 126]. Accordingly, this study and others have noted that the MSN module is enriched with AD genetic risk factors, suggesting it maintains a causal role in disease pathogenesis [62, 64, 74, 96]. The precise mechanisms employed by the MSN module are unclear, though many of its microglial proteins have known roles in phagocytosis, and module ontology analyses have demonstrated strong associations with glucose metabolism [96].

In addition to this early neuroprotective module, the AD network proteome features a second inflammatory module co-enriched with microglia, astrocytes, and endothelial cell type markers that is consistently elevated later in disease. This module contains extracellular matrix (ECM) proteins responsible for mediating cell–cell interactions, with its most preserved hubs including scaffolding (CAV1) and collagen proteins (COL6A1, COL6A3). The co-expression of glial and endothelial proteins supports the role of vascular-mediated inflammation in AD pathogenesis. The growing depth and complexity of proteomic networks may result in modules more specific to the endothelium and better define this vascular biology. We recently performed an unbiased network analysis on a remarkably deep AD brain proteome (DLPFC) of >8000 quantified proteins generated by TMT-MS coupled with off-line fractionation [63, 75]. Among the resulting 44 modules, we resolved an ECM-associated module with highly significant endothelial enrichment and relatively weak glial co-expression. This module was strongly associated with wound healing and other aspects of humoral immunity and featured modest upregulation in AD dementia. While these findings further imply a vascular contribution to AD pathogenesis, such disease-related alterations in vascular proteins could also reflect the impact of atherosclerosis or other cerebrovascular risk factors on the AD proteome [107].

### A link between oligodendrocytes and AD risk

The AD brain network proteome also features conserved upregulation of oligodendrocyte-enriched modules associated with myelination (Fig. 1). Comprising ~75% of all glial cells in the brain, oligodendrocytes generate and maintain the lipid-rich myelin sheaths that insulate neuronal axons and facilitate signal transmission [12]. Despite their abundance, oligodendrocytes are perhaps the least studied of non-neuronal brain cells in the context of AD. Yet, a growing body of evidence suggests that myelin dysfunction is an early and essential component of AD pathogenesis. For instance, white matter lesions attributable to aberrant myelin breakdown have been widely reported in the brains of individuals with mild cognitive impairment (MCI) [127]. Furthermore, magnetic resonance imaging (MRI) data indicate that individuals with the risk allele *APOEε4* have accentuated myelin breakdown in AD [128, 129]. This association with genetic risk and early disease indicates that myelin dysfunction may be an inciting event in the pathogenesis of disease.

Accordingly, proteomic network analyses of the AD brain have demonstrated modest upregulation of a highly conserved oligodendrocyte-enriched myelination module in both asymptomatic and symptomatic disease [62–64, 74, 96]. In addition, a handful of studies have found that like the MSN module, this myelin module features over-representation of AD GWAS candidates, suggesting a causal role for oligodendrocyte processes in disease [62, 96]. In an examination of *APOE* genotype on the brain network proteome, Johnson et al. found that the most significant effect was exerted by the *APOEε2* allele on the oligodendrocyte module, suppressing its disease-related changes in those with AD [96]. Dai et al. also observed this *APOEε2* effect on oligodendrocyte and other cell type markers in a separate network proteome of the AD brain [104]. These associations between AD risk and oligodendrocyte function mirror the results of a recent large-scale transcriptomic network analysis in the human AD brain (DLPFC, visual cortex, cerebellum), which demonstrated significant enrichment of AD GWAS candidates in conserved disease-associated oligodendrocyte-enriched modules, including *BIN1, PICALM*, and several others [130]. In a comparison of this transcriptome network to an independently constructed proteomic network from AD brain tissue, the authors found at least 50% overlap between oligodendrocyte modules of the two datasets. Additional transcriptomic network analyses have similarly found enrichment of genes associated with either AD risk or Aβ processing among oligodendrocyte-specific modules [131, 132].

The mechanisms by which oligodendrocyte dysfunction contribute to AD pathogenesis remain unclear. In an intriguing network proteomic analysis of over 400 brains (DLPFC), Wingo et al. found that cerebral atherosclerosis may be the missing link between oligodendrocytes and AD dementia [107]. In this study, cerebral atherosclerosis was pathologically assessed by visual inspection of Circle of Willis vessels and proximal branches and graded independently of gross infarcts, microinfarcts, or other white matter lesions. The oligodendrocyte-enriched myelin module was one of only two protein communities in the co-expression network associated with both cerebral atherosclerosis and AD dementia, independent of Aβ, tau tangles, infarct burden, and a variety of other pathologies. These associations also persisted after adjustment for hypertension, diabetes, and other cerebrovascular risk factors. In addition, the authors observed no similar statistical correlations between cerebral atherosclerosis and Aβ accumulation or tau burden. Overall, these findings suggest that cerebral atherosclerosis, a common occurrence among the elderly, contributes to AD through oligodendrocyte dysfunction and injury to the myelin sheath, regardless of hallmark proteinopathy, gross ischemic disease, or cerebrovascular risk factors.

### A role for RNA binding in the biochemical phase

Modules enriched with RNA binding and splicing proteins are also consistently upregulated in the AD network proteome (Fig. 1). In contrast to the inflammatory and myelination modules, these RNA-binding/splicing modules are not enriched with glial or other cell type-specific markers. In a deep proteomic network analysis of control, AsymAD, and AD DLPFC tissues, Johnson et al. identified several AD-associated RNA-binding/splicing modules notably increased in asymptomatic and symptomatic AD. The disease-associated alterations in these protein modules were independent of cell type, persisting even after the dataset was regressed for cell type-specific effects [74]. The authors subsequently concluded that RNA-binding proteins may drive the proteopathic “biochemical” phase of AD.

These findings correlate well with previous analyses of the AD brain detergent-insoluble proteome, which have repeatedly demonstrated the association of U1 small nuclear ribonucleoproteins (U1 snRNPs) and other RNA-binding proteins with tau tangles [133–137]. These ribonucleoproteins, constituents of the spliceosome complex responsible for RNA processing, are mislocalized in AD to the cytoplasm of neuronal cell bodies where they aggregate with NFTs and contribute to widespread alterations in RNA processing [134]. Consistent with network proteomic findings, these aggregated U1 snRNPs have been observed in asymptomatic AD brains [135], providing further support for aberrant RNA processing in early disease. Interestingly, this aggregation of U1 snRNPs with tau appears to be unique to the AD brain and absent among other primary tauopathies, such as FTLD [134, 138].

In a recent study of U1-70K, an snRNP consistently associated with NFTs in the AD brain, Bishof et al. utilized a network-based proteomic approach to functionally characterize the co-immunoprecipitated interactome of U1-70K and identify those protein–protein interactions most dependent on U1-70K domains implicated in aggregation and AD pathogenesis [138]. This analysis successfully organized the U1-70K interactome into seven biologically meaningful modules, two of which demonstrated a strong association with pathogenic U1-70K domains. These two modules, both linked to RNA splicing ontologies, were enriched in proteins with structural and aggregation properties similar to those of U1-70K that could serve as additional AD biomarkers, such as pre-mRNA splicing factor LUC7L3. These findings underscore the utility of unbiased network-based analysis in the characterization of protein–protein interactions and their dependence on sub-molecular domains.

In contrast to cell type-enriched modules, RNA-binding/splicing modules demonstrate limited overlap with the transcriptomic network of the AD brain. This observation suggests that global protein-level analyses will maintain a uniquely valuable role in unraveling the proteopathic biochemical phase of AD. In addition to RNA-binding/splicing modules, network proteomics has revealed one other highly conserved AD-associated module with possible links to this phase of disease. Unlike the RNA-binding/splicing modules, this module is consistently downregulated throughout AD and strongly associated with the cytoskeleton (Fig. 1) [62, 63, 96]. Its hub proteins often include tubulin subunits or tubulin-associated proteins, such as dynactin subunit 1 (DCTN1). As microtubule stability is one emerging means of targeting tau pathology [49], this module may also yield promising disease-associated biochemical phase markers for therapeutic targeting.

### A link between synaptic and mitochondrial dysfunction

It is well-known that synaptic loss is one of the strongest correlates of cognitive decline in AD, even more so than Aβ plaques and tau tangles [139]. While neuronal death contributes substantially to this synaptic decline, it is increasingly clear that other mechanisms may also play a role in AD synaptic dysfunction. A growing body of evidence in mouse models indicates alterations in synaptic networks, prior to the onset of gross neuronal loss in the cerebral cortex [140, 141]. This is reflected in the global AD network proteome, which consistently demonstrates the progressive downregulation of synaptic modules in asymptomatic and symptomatic AD brains, a finding that appears to be preserved across various cortical brain regions [62, 96, 105]. In addition, emerging cerebrospinal fluid (CSF) proteomic data has revealed alterations in a number of synapse-associated proteins in preclinical disease [63]. The mechanism underlying these early synaptic protein changes remains unclear, with possibilities including morphological remodeling, regional changes in distribution, and immune-mediated synaptic pruning, among others. Furthermore, it has yet to be determined whether these preclinical synapse changes represent a homeostatic, protective, or detrimental event in AD pathogenesis.

In a proteome-wide association study of nearly 150 subjects, Wingo et al. used co-expression network analysis to demonstrate the increased abundance of synaptic proteins in the postmortem brain tissues (DLPFC) of individuals with antemortem stable cognitive trajectories, regardless of the burden of Aβ plaques or tau tangles [106]. This suggests that the downregulation of synaptic proteins in AsymAD may ultimately prove detrimental to cognitive stability, promoting conversion to symptomatic disease. Interestingly, this study also found heightened levels of mitochondrial proteins among cases with preserved cognition, perhaps reflecting the close relationship between neuronal synapses and mitochondria. Synapses are markedly enriched with energy-producing mitochondria, which fuel both pre- and post-synaptic signaling processes. Therefore, mitochondria are critical for maintaining synaptic integrity and function, explaining why an over-abundance of both module types may be observed in individuals with high levels of cognitive resilience. In a more recent proteomic analysis, Yu et al. paralleled these network results, identifying associations between cognitive resilience and cortical proteins involved in synaptic, metabolic, and neurogenic functions [142].

Overall, the AD brain network proteome suggests close links between synaptic and mitochondrial modules. It is not uncommon for modules linked to synaptic ontologies to feature a variety of metabolic proteins [63]. As demonstrated in Fig. 1, ATPase subunits can be found among the preserved hubs of both synaptic and mitochondrial modules. As discussed later, this close link between synaptic and metabolic protein expression is also reflected in CSF, which features stark elevations in both types of proteins in the asymptomatic stages of disease [63]. These early CSF protein derangements suggest the aberrant exocytosis of neuronal components into the extracellular space, which could be explained by synaptic pruning. Emerging evidence in AD mouse models indicates this homeostatic, microglia-mediated synaptic phagocytosis may be one of the earliest events in AD progression and contribute to synaptic losses during asymptomatic stages of disease [143]. This mechanism would also explain, at least in part, the glial protein elevations observed in the early AD brain, providing a mechanistic framework for a variety of proteomic network changes during the cellular phase of disease.

## Emerging technologies for cell type-specific proteomics

**Fig. 2:**
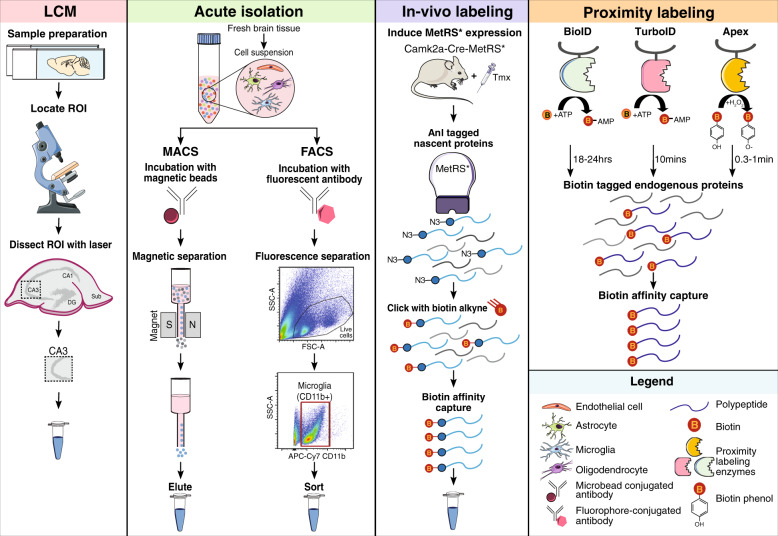
Local proteomics approaches to define the cellular basis of Alzheimer’s disease. Summary of approaches: Laser capture microdissection (LCM) is a method that can procure subpopulations of cells or very small regions of interest under direct microscopic visualization. LCM (pink panel) has been used to micro-dissect neurons from both fresh frozen human brain and formalin-fixed paraffin embedded tissue sections for proteomic analyses. Prior to dissection, immunohistochemical or histological staining of fixed tissue sections is performed to identify a specific cell type or population of cells in a region of interest without compromising protein quality. For acute isolation of brain cell types (green panel), a fresh brain sample needs to be processed to yield a single-cell suspension which is then subjected to magnetically-activated cell sorting (MACS) or fluorescent-activated cell sorting (FACS). For MACS, the desired cell type is labeled with a 50 nm magnetic microbead conjugated to an antibody specific to cell-surface receptor. After incubation, the sample is placed on a magnet to drain unbound cells and retain desired cell type within the column. Once the column is removed from the magnet, the bound cells are released and collected for downstream analyses. Analogous to MACS, in FACS, the single-cell suspension is incubated with a fluorophore-conjugated antibody specific to a cell-surface receptor. Subsequently, the desired cell type is sorted based on their size and fluorescent signal directly into a buffer amenable for downstream proteomic analyses. In vivo biorthogonal amino acid tagging (BONCAT) of proteins (purple panel) is achieved by expressing MetRS* under a cell type-specific promoter in a mouse (Camk2a-Cre-MetRS*). MetRS* harbors a mutation (L247G) in the amino acid binding site which preferentially tags nascent proteins with an azide-tagged methionine analog, azidonorleucine (Anl). After treating the mice with tamoxifen (Tmx) to facilitate Cre-mediated recombination, Camk2a cells express MetRS* and nascent proteins are tagged with Anl. The azide residue of Anl is amenable to copper-catalyzed azide-alkyne cycloaddition or “click” chemistry. Following protein extraction, Anl-tagged proteins residues are “clicked” with a PEG-biotin-alkyne and then purified using avidin beads or avidin resin for subsequent MS analyses. Proximity labeling (orange panel) is achieved by various enzymes that biotinylate proximal endogenous proteins. The BioID approach uses the *Escherichia coli* biotin ligase, BirA*, with a catalytic site mutation (R118G). The mutation destabilizes the enzyme, facilitating active biotin molecules (biotinoyl-5′-AMP) to dissociate and bind to primary amines of exposed lysine residues on adjacent proteins. APEX catalyzes the oxidation of biotin-phenol to the short-lived (<1 ms) biotin-phenoxyl radical in the presence of hydrogen peroxide, which then reacts with electron-rich amino acids, such as tyrosine, in neighboring proteins. TurboID was developed by taking advantage of yeast display-based directed evolution. TurboID retains the promiscuous biotinylation property of BioID, but rapidly labels proteins in 10 min compared to the 18–24 hrs by BioID. Subsequent to biotin labeling and protein extraction from a sample, proteins can be affinity captured by streptavidin beads or matrices for downstream proteomic analyses by MS.

Proteomic profiling of human brain tissue has indirectly revealed potential cell type-specific mechanisms of AD. The next challenge for proteomic research is gaining the cellular and temporal resolution to further define the causative role of cell-mediated dysfunction in AD pathogenesis. In this section, we review several approaches for localizing the AD proteome to a brain region or cell type to advance our understanding of the cellular phase of disease (Fig. 2). Cell type-specific proteomics in the human brain is currently in its infancy, primarily due to the inability to isolate live, pure cell populations from frozen brain and limited access to fresh postmortem tissue. Thus, extending localized proteomic approaches to mouse models of AD pathology (Table 1) will be critical for confirming the network architecture and investigating causal molecular changes during the cellular phase of disease. Furthermore, cell type-specific proteomics in disease and aging mouse models can serve to de-convolute complex human brain data and provide cellular-level resolution to peripheral biomarkers. In addition, this work will significantly enhance our understanding of the aging-dependent course of cell type-specific perturbations, thus allowing us to pinpoint possible opportunities for therapeutic intervention and enhance biomarker development.

### MS-coupled laser capture microdissection

Laser capture microdissection (LCM) is a method that can procure subpopulations of cells or very small regions of interest under direct microscopic visualization (Fig. 2). LCM has been used to micro-dissect neurons from both fresh frozen human brain and formalin-fixed paraffin embedded tissue sections for proteomic analyses [144, 145]. Prior to dissection, immunohistochemical or histological staining of fixed tissue sections is performed to identify a specific cell type or population of cells in a region of interest. Protein yield for LC-MS/MS analysis is not impacted, suggesting that LCM-based approaches are very powerful and underutilized for cell type-specific proteomics of archival human brain samples. Drummond et al. [144] used LCM to comprehensively characterize the protein composition of Aβ plaques microdissected from the hippocampus of two AD subtypes: rapidly progressive AD and sporadic AD. LCM coupled to LFQ-MS of these samples revealed proteins increased in rapidly progressive AD plaques (e.g., α-synuclein) to be decreased or have no known involvement in sporadic AD [144]. This study is a powerful example of how an unbiased, localized proteomics approach can further our understanding of proteins involved in AD pathogenesis that would otherwise be diluted in a global proteomics analysis of bulk tissue. Due to recent technological advancements (e.g., optical resolution), LCM can capture areas or cells of interest with much higher precision and speed, though the time it takes to do the dissection is subjective (Table 2). In addition, LCM-based MS results in a lower proteome coverage compared to bulk brain proteomics, and the inability to confidently exclude contamination by cells that are not of interest poses a challenge (Table 2).

**Table 2:** Summary of the advantages and disadvantages of local proteomics approaches.

Approach	Advantages	Disadvantages
Laser capture microdissection (LCM)	• Ability to image cell type and structure and acquire cell count • A wide range of tissues can be used: unfixed frozen postmortem brain tissue, formalin-fixed paraffin embedded brain tissue, hematoxylin & eosin stained or immunostained tissues • One tissue section can be dissected several times for different regions • Dissection does not disturb cells’ molecular state • Area size of 1.5 mm^2^ is sufficient for MS	• Tissue drying during dissection • Dissection process can be time-consuming: 5 min to 8 hrs depending on the size, cell type, and number of areas or cells to be collected • Inability to confidently exclude cells that are not of interest • Protocol not optimized for smaller, non-neuronal cell types or single cells • Lower proteome coverage compared to bulk brain proteomics
Magnetic-activated cell sorting (MACS)	• High throughput • High purity • Selective and rapid method • Enrichment can be scaled up or down to desired yield	• Contamination by acellular debris or unbound cells • Immunomagnetic beads might cause mechanical shear • Sequential isolations significantly reduce yield • Cannot isolate intact neurons
Fluorescence-activated cell sorting (FACS)	• High sensitivity, throughput, and purity • Isolate multiple cell types simultaneously based on immunopheno type alone • Sort complex cell types with multiple markers • Separate cells based on cell size, density, and morphology, cell cycle status, intracellular cytokine expression, and metabolic profile • Capture immunophenotyping data for 12 surface epitopes • Minimum of 12,000 cells are sufficient for MS	• Long isolation procedure (3+ hrs) • Shear stress from the FACS instrument • Slow sorting process – depends on number of cell populations that need to be collect • Recovery is 50–70% on most sorters, need a high number of cells at the beginning • Fluorophore spillover into non-specific channels between cells with closely related immune phenotypes • Cannot isolate intact neurons
Bio-orthogonal non-canonical amino acid tagging (BONCAT)	• Identification of low abundance, low copy number newly synthesized proteins with higher magnitude • Click chemistry procedure is modular and relatively simple • Lineage tracing of proteins • Established transgenic mouse line under the Cre/Lox system	• Cost of Anl and special diet for mouse studies • Obtaining a good signal-to-noise ratio between endogenously biotinylated proteins and biotin clicked Anl-tagged proteins • Depth of proteome is lower than traditional proteomics • Reduced labeling efficiency due to competition between endogenous MetRS and MetRS*
Proximity labeling (BioID, APEX, TurboID)	• Rapid kinetics of biotinylation without click chemistry • Detect weak or transient protein interactions as well as soluble and insoluble proteins • Ready bioavailability of biotin in the brain after peripheral administration • Acquire a more global proteome unlike the nascent proteins in BONCAT	• Noise introduced by endogenous biotinylation • APEX approach is limited to in vitro experiments since biotin-phenol is toxic • TurboID can sequester endogenous biotin and cause toxicity • Saturation of proximal labeling sides with prolonged biotin supplementation • Lack of mouse models for BioID and TurboID approaches

### Acute isolation of brain cell types

Unlike transcriptomic studies that can be performed on intact nuclei from frozen brain (mouse or human), proteomic analyses of brain cell types require isolation of intact cells from the brain, for which fresh unfrozen brain is a pre-requisite. Several strategies exist to isolate the cell type(s) of interest in a highly pure form with minimal contamination by other cells or acellular elements.

#### Magnetic-activated cell sorting (MACS)

MACS aims to facilitate the rapid, high-throughput, immunomagnetic separation of a pure cell type population from a heterogeneous population (Fig. 2). The MACS approach has been successfully applied to enrich four brain cell types (neuronal progenitors, microglia, astrocytes, and oligodendrocytes) from adult mice for high-resolution MS-based proteomics [97]. This in-depth analysis resulted in the largest assortment of cell type-resolved proteomic data of the brain and is frequently used for cell type enrichment analyses [97]. Recently, quantitative TMT-MS was performed on acutely isolated CD11b^+^ MACS-enriched microglia from adult (6–7 mo) mice of normal, acute neuroinflammatory (lipopolysaccharide (LPS)-treatment), or chronic neurodegenerative (5xFAD model) states [146]. Of 4133 proteins identified, 187 microglial proteins were differentially expressed in 5xFAD mice, including proteins with known (e.g., Apoe) and novel (e.g., Cotl1) relevance to AD biology. Cotl1 was identified as a novel microglia-specific marker with increased expression and strong association with AD neuropathology. Apoe protein was also detected within Aβ plaque-associated microglia, suggesting a role for Apoe in phagocytic clearance of Aβ. Several proteins increased in human AD brain were also upregulated by 5xFAD microglia (e.g., Aβ peptide) [146]. This deep and comprehensive proteomic study of isolated mouse microglia revealed shared neuroinflammatory disease mechanisms between mouse models of AD pathology and human AD, emphasizing the value of state-of-the-art proteomics methods for resolving cell type-specific contributions to disease [146]. One key strength of the MACS is the ability to rapidly isolate cells of interest with relatively high purity without dependence on cell sorters; however, despite high cellular purity, MACS suffers from contamination by acellular debris or unbound cells (Table 2) [147].

#### Fluorescence-activated cell sorting (FACS)

FACS is another highly sensitive and high-throughput procedure for isolating cells from a heterogeneous population (Fig. 2). Fluorescent-conjugated antibodies against cell-surface epitopes are used to label cell type(s) of interest. The fluorescently labeled cells can be sorted directly into a buffer amenable for LC-MS/MS (Fig. 2). The FACS approach has been applied for transcriptomic studies [148–152] and proteomic applications are now gaining momentum. A recent study compared the proteomic profiles of MACS-enriched microglia and FACS-isolated microglia from adult mouse brain and found that the FACS-isolated microglia proteome was significantly enriched in canonical microglial proteins (e.g., Ctsd) and contained much lower levels of non-microglia proteins (e.g., Gfap) [153]. Also, FACS coupled to LC-MS/MS was employed to characterize neuronal and non-neuronal nuclear proteomes. Intact nuclei were purified from frozen human brain tissue and sorted based on the expression of a neuron-specific splicing factor, NeuN. Comparative analysis of NeuN-positive and NeuN-negative nuclear proteomes revealed a number of transcription and splicing factors not previously known to be expressed in a cell type-specific manner in human brain [154]. This method provides a unique opportunity to identify cell type-specific nuclear proteins, as well as histone modifications and regulation networks, that may be altered in AD. FACS is an ideal method for simultaneously sorting multiple cell types based solely on immunophenotype. Recently, a cell isolation methodology termed concurrent brain cell type acquisition (CoBrA) was used to isolate microglia, endothelial cells, astrocytes, and oligodendrocytes from mouse brain for RNAseq studies [152]. This FACS pipeline can be easily optimized for proteomic analyses of multiple cell types with >95% purity by confidently excluding debris. Moreover, utilizing FACS for high-throughput single-cell proteomics by MS (SCoPE-MS) [155], a fairly new method, holds great promise and potential for integration with single-cell RNAseq. Yet, FACS is not without limitations (Table 2). The prolonged handling of live cells for FACS and the shear stress imposed on them by the sorter can spuriously activate sensitive cells such as microglia. One inherent limitation of MACS and FACS is that live neurons cannot be sampled from adult brain, tremendously limiting our ability to understand neuron-specific proteomic changes occurring in vivo, as described below. Realistically, both approaches can be considered complementary and their relative strengths and weaknesses (Table 2) should guide rigorous study design with appropriate controls.

### In vitro and in vivo protein labeling strategies

#### Bio-orthogonal non-canonical amino acid tagging (BONCAT)

Proteins within a specific cell type can be uniquely labeled in vivo via BONCAT (Fig. 2) and isolated from a complex tissue, such as the brain, for MS-based proteomics. BONCAT leverages the L274G mutant of mouse methionine-tRNA synthetase (MetRS*) to tag newly synthesized “nascent” proteins with an azide-tagged methionine analog, azidonorleucine (Anl) [156–158]. Anl residues are “clicked” with a PEG-biotin-alkyne and then purified using avidin beads or avidin resin for subsequent MS analyses. Recently, BONCAT was utilized to characterize excitatory and inhibitory neuronal proteomes in adult mice [158, 159]. MetRS* knock-in mice were crossed with Camk2a-Cre-ert2 mice followed by tamoxifen treatment to induce Cre-mediated recombination. Consequently, only Camk2a cells expressed MetRS* and could incorporate Anl into their proteome. Brain homogenate from these mice underwent click chemistry, affinity purification, and MS to obtain the proteomic profile of excitatory hippocampal neurons. The Camk2a hippocampal proteome was significantly enriched with proteins represented by neuronal components, such as synaptic transmission and synaptic plasticity. Several key proteins (e.g., APP, Grm5) linked to neurodevelopmental or neurodegenerative disorders were also identified [158]. Interesting data from this study showed that when mice were exposed to an environment with enriched sensory cues, there was a change in the neuronal proteome, which represented a response or adaptation to the external stimuli. The fidelity of in vivo BONCAT has thus far only been shown in mouse excitatory neurons; however, it represents a highly promising new strategy to derive nascent proteomes from heterogeneous brain tissue in a cell type-specific manner. Depth of proteome coverage, labeling efficiency, and obtaining a good signal-to-noise ratio between endogenously biotinylated proteins and biotin clicked Anl-tagged proteins are important factors to take into consideration when using this approach (Table 2).

#### Proximity labeling

Proximity labeling with enzymes is another strategy to achieve cell type-specific global proteomic labeling [160]. The enzymes catalyze the formation of reactive biotin species, which diffuse out of the active site to biotinylate proximal endogenous proteins (Fig. 2). Subsequently, biotinylated proteins can be enriched through affinity capture and characterized via MS. There are three proximity labeling technologies: BioID, APEX, and TurboID. The BioID approach uses the *Escherichia coli* biotin ligase, BirA*, to biotinylate proteins within ~10 angstroms [161]. BioID has been used extensively in vitro [162–166] to define interacting protein partners and proteins in subcellular compartments, but its application in vivo is limited. By fusing BirA* to a bait protein with a synaptic localization signal or nuclear pore signal, BirA* expression was specifically guided to the synapse or nuclear pore complex to achieve biotinylation of the respective proteomes [167, 168]. More recently, BioID was used to effectively label proteins within flies (*Drosophila melanogaster*) or worms (*Caenorhabditis elegans*) [169]. Similar to the BioID approach, an engineered ascorbate peroxidase, or APEX, could also be used for efficient proximity labeling of proteins [160] (Fig. 2). With APEX, researchers have been able to profile the mitochondrial matrix proteome and characterize the structure of a mitochondrial uniporter [170, 171]. A major advantage of APEX over BioID, is the rapid kinetics and high efficiency of labeling; however, APEX is not suitable for in vivo studies due to the toxicity of biotin-phenol. A rapid and non-toxic labeling technology, TurboID, was developed by taking advantage of yeast display-based directed evolution. TurboID retains the promiscuous biotinylation property of BioID, but rapidly labels proteins in 10 minutes compared to the 18–24 hours required by BioID (Fig. 2). TurboID-based proteome labeling has been successfully applied in flies and worms without much toxicity and with high labeling efficiency [169]. However, the levels of endogenous biotinylation in the rodent brain is a disadvantage of in vivo proximity-dependent biotinylation strategies (Table 2). This can be partly overcome by omitting regions with known elevated levels of endogenous biotinylation or subtracting out known endogenously biotinylated proteins from proteomic analyses. One significant advantage of these approaches (Table 2) is the ability to capture weak or transient protein interactions as well as soluble and insoluble proteins because of the high affinity of biotin for avidin, a challenge often faced with the use of classic antibody-based affinity purification/MS methods.

## Translating the brain network proteome into AD biomarkers

We have outlined the proteomic evidence showcasing AD as a vastly complex, multi-system disorder that extends beyond “hallmark” pathology to include a variety of cellular mechanisms with potentially central roles in disease progression. Yet, the intricate pathology observed among meticulously categorized autopsy research cohorts likely reflects only a fraction of the vast biological complexity found in clinical AD populations. It is becoming increasingly clear that most demented elderly individuals harbor more than one “hallmark” dementia pathology [37]. Indeed, up to 90% of individuals with the classic amyloidosis of AD may feature concurrent vascular disease, TDP-43, or other degenerative pathologies [39]. These high rates of overlapping pathologies combined with additional biological heterogeneity introduced by individual genetic and environmental factors make for an overwhelmingly complex pathophysiological landscape among the over 40 million individuals thought to be living with AD worldwide.

Our current diagnostic framework for clinical AD fails in capturing this biological diversity. The limited number of clinically established AD biomarkers, such as amyloid PET imaging and CSF Aβ and tau levels, reflect only “hallmark” pathology. Such a reductionist approach to AD stands to hinder advancements in diagnostic subtyping, disease monitoring, and therapeutic development. Approaching such a biologically heterogenous disease as a single entity in clinical trials may account for many of the AD drug failures we have encountered to date. Thus, AD requires a new diagnostic framework, one reliant on more diverse biomarker assays reflective of a wide range of pathophysiology. A tool of this nature could not only allow for detailed biological subtyping, but also usher in a new age of patient-tailored therapeutics in neurodegeneration. For these reasons, systems-based approaches to biomarker discovery, as championed by the AMP-AD initiative, are growing in popularity among neurodegenerative disorders. In the following section, we describe early efforts to translate the network AD brain proteome into systems-based, physiologically diverse, multiplex biomarker assays capable of advancing our clinical framework of disease.

### Integration of brain and biofluid proteomes

**Fig. 3:**
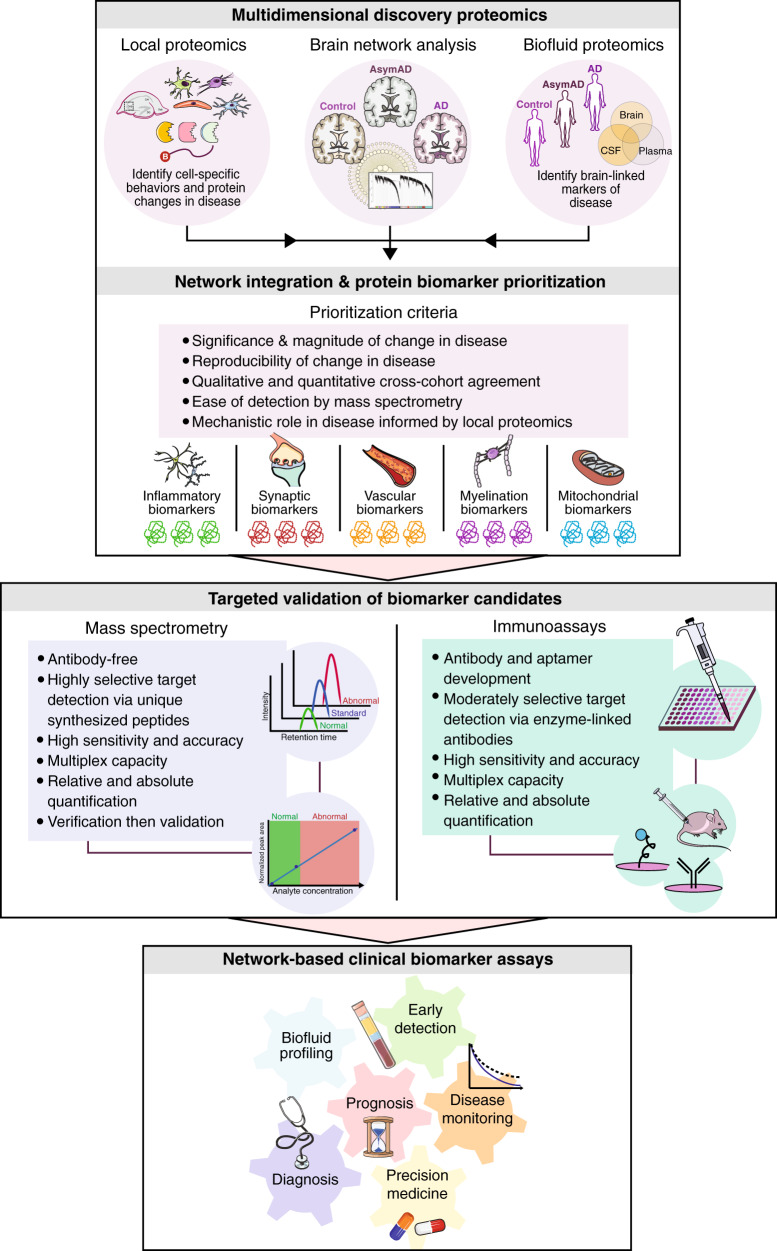
Conceptual framework for translating brain protein networks into clinical biomarkers. In this framework, multidimensional discovery-driven proteomics data collected from local cell type-specific approaches and antemortem biofluids will be integrated with the AD brain network proteome to identify systems-based panels of promising CSF and/or plasma biomarkers. Target prioritization will rest on the significance, magnitude, reproducibility, and ease of detection of the candidate biomarker in disease. Prioritized targets will also require links to disease mechanisms, informed in part by localized and cell type-specific proteomics. These network-based biomarker panels will then be validated using targeted quantitation approaches, including mass spectrometry (MS) and immunoassays. Both validation methods offer highly sensitive and accurate quantitation, though MS may offer certain advantages, such as highly selective target detection using unique peptides and the ability to cost-effectively analyze large panels of proteins in an initial verification phase prior to more expensive validation efforts. Validated biomarker panels representing a wide range of pathophysiologies could serve a variety of clinical uses, including preclinical profiling, disease monitoring, measuring therapeutic response, and confirming target engagement.

To increase the pathophysiological diversity among CSF biomarkers of AD, we have developed a novel approach that integrates the global AD brain network with proteomic analysis of CSF (Fig. 3). The direct proximity of CSF to the brain presents a strong rationale for discovery-driven integration of these two proteomes. Johnson et al. recently found that ~20 proteins from the highly conserved MSN module demonstrated significant elevations in AD CSF [96]. These CSF protein elevations were consistent across two different AD cohorts comprising a total of nearly 400 spinal fluid samples. As discussed previously, the anti-inflammatory microglia-enriched MSN module is elevated in the AsymAD brain and thought to play a causative role in disease pathogenesis. The reflection of such disease-associated cellular processes in the CSF could provide a means of detecting and monitoring treatment responses.

In another recent large-scale study, Higginbotham et al. used an integrative proteomic approach to examine the statistical overlap of the AD brain network proteome (DLPFC) with differential expression in the AD CSF proteome [63]. Fifteen of the 44 brain modules identified in this study strongly overlapped with the CSF proteome. These 15 brain modules were also high-yield sources of markers differentially expressed in AD CSF, collectively harboring nearly 300 proteins with significantly altered levels in AD spinal fluid compared to controls. Based on their corresponding brain modules, these ~300 CSF AD targets were then segregated into five systems-based biomarker panels representing a wide range of brain pathophysiology, including synaptic transmission, vascular biology, myelination, glial-mediated inflammation, and energy metabolism. Using high-throughput TMT proteomic analysis, proteins from these five panels were validated in multiple additional CSF cohorts, totaling >500 spinal fluid samples. The results of these validation studies were then used to narrow down these biomarker panels from roughly 300 to 60 proteins across the five panels. Target prioritization was based on a variety of criteria, including significance and magnitude of change in disease, reproducibility of these disease-related alterations, and the ease of mass spectrometry detection and quantification (Fig. 3). Interestingly, the final CSF marker panels demonstrated AD-specificity and altered levels in AsymAD spinal fluid, indicating a potential role for these panels in preclinical disease stages. In two separate smaller analyses of AD CSF, many of the same AD-associated synaptic and metabolic biomarkers were identified, further supporting the reproducibility these systems-based CSF marker panels [78, 172].

Plasma biomarker discovery represents another potential extension of AD brain network analysis. The rationale for using plasma biomarkers as proxies for brain processes [173] has been demonstrated for multiple neurodegenerative diseases [174, 175]. While targeted proteomic approaches have been primarily used to measure plasma Aβ [176, 177] and phosphorylated tau [178, 179], recent improvements in MS technology and chromatography have renewed interest in discovery plasma proteomics in AD [180]. For example, discovery TMT proteomics was recently applied to plasma samples of two independent cohorts to predict Aβ burden in preclinical disease with high accuracy [181]. Moving forward, integrative analyses that successfully correlate proteomic signatures across brain, CSF, and plasma will prove key for the advancement of ideal network-based biomarkers of disease (Fig. 3).

### Targeted validation and assay development

Following discovery-driven proteomics, targeted assays are typically employed in large numbers of clinical samples to validate specific candidate biomarkers or multiplexed biomarker panels (Fig. 3). Antibody- or aptamer-based immunoassays are often used as a strategy for target validation [182, 183]. These assays are capable of high-fidelity validation, versatile in application, and easier to execute than MS-based assays. However, immunoassay validation can present certain challenges. First, the costs of commercially available antibody or aptamer reagents necessary for large-scale studies of hundreds or thousands of proteins in sizeable clinical research studies can be prohibitive, limiting validation and translation efforts. Second, in the absence of high-quality, commercially available antibodies or aptamers, assays must be generated de novo, optimized, and validated separately, a process that can also be costly and time-consuming. Finally, immunoassay approaches are often unable to specifically detect biologically or pathologically relevant isoforms or PTMs of a protein.

As an antibody-free platform with robust sensitivity, high accuracy, and an exceptional multiplex capacity, targeted MS technologies such as SRM/MRM and PRM offer a promising alternative to the challenges of immunoassay validation [184–187]. As Cilento et al. argue in a recent review of targeted MS in AD biomarker discovery, these approaches provide a cost-effective means of systematically verifying large panels of protein biomarker candidates prior to more expensive and large-scale validation testing [187]. This workflow typically begins with the synthesis of unique and protein-specific peptides to facilitate the direct detection of candidate biomarkers and development of multiplex assays. These targeted MS assays, which are capable of measuring hundreds of peptide biomarkers simultaneously, are then applied to a small set (~10–50) of patient samples and critically analyzed for reproducibility and assay adaptability. Once verified, a smaller panel of the most promising candidates can enter the validation stage, in which they are analyzed in several hundred (~100–500) samples to critically assess sensitivity and specificity. This validation analysis represents the final preclinical stage, after which chosen candidates can be further investigated for clinical application [187, 188].

Targeted MS approaches have been increasingly employed for the detection and quantification of protein biomarkers from biofluids [187]. Several studies have successfully used targeted MS strategies to quantify Aβ, tau, and APOE protein levels in CSF with similar disease-association efficacy to that of traditional ELISA assays [189–191]. More recently, targeted MS was applied to a more diverse panel of promising AD CSF biomarkers reflecting a much wider array of disease pathophysiologies [192]. In this study, Zhou and colleagues used PRM to quantify a panel of brain-derived proteins significantly altered in a discovery-driven proteomic analysis of AD CSF. Several proteins, including tau and neurofilaments, were significantly increased in AD CSF and capable of distinguishing AD cases from controls and non-AD dementia. Protein targets mapping to synaptic, metabolism, and neuroinflammation modules in brain [96] were quantified with high precision, showcasing their potential for future biomarker translation. SMOC1, YWHAZ, ALDOA, and MAP1B emerged as biomarkers that could best discriminate between individuals with AD and non-AD cognitive impairment, correlating well with Aβ and tau levels [192]. Overall, these results further illustrate the utility of targeted MS strategies to significantly advance AD biomarker discovery toward network-based protein assays with multiple clinical uses in disease (Fig. 3), including preclinical profiling, disease monitoring, measuring therapeutic response, and confirming target engagement.

## Future directions

In summary, global network proteomics has revealed a highly reproducible and holistic window into the complex biochemical and cellular alterations in the brains of individuals with asymptomatic and symptomatic AD. As the network landscape of the AD proteome continues to emerge, future directions will include the (a) exploration of local cell type-specific proteomes to better elaborate the disease mechanisms implicated in these global studies, (b) integration of these brain networks and hubs with protein analyses of CSF and plasma, and (c) large-scale verification and validation of the most promising brain-linked biomarkers in additional human samples using targeted proteomic strategies. This validation will also include the longitudinal measurement of promising biomarkers in large cohorts, such as the Alzheimer’s Disease Neuroimaging Initiative (ADNI) and the Dominantly Inherited Alzheimer Network (DIAN), to define their reactivity to disease progression and treatment. Together these efforts promise to not only expand our understanding of AD pathogenesis, but also fulfill many unmet clinical needs in AD diagnostics, disease monitoring, and therapeutics.

## Funding and disclosure

Research reported in this publication was supported by the National Institute on Aging of the National Institutes of Health (F32AG064862, K08-NS099474-1, R01 NS114130-01A1, R01AG053960, R01AG057911, R01AG061800, RF1AG057471, RF1AG057470, RF1AG062181), the Accelerating Medicine Partnership for AD (U01AG046161 and U01AG061357), the Emory Alzheimer’s Disease Research Center (P50AG025688), and the NINDS Emory Neuroscience Core (P30NS055077). Additional support was provided by grants from the Alzheimer’s Association (Award no. 37102 to S. Rangaraju), Foundation for the National Institutes of Health (FNIH), Alzheimer’s Research UK (ARUK), and the Weston Brain Institute (11060). The authors declare no competing interests.
